# Ontology integration to identify protein complex in protein interaction networks

**DOI:** 10.1186/1477-5956-9-S1-S7

**Published:** 2011-10-14

**Authors:** Bo Xu, Hongfei Lin, Zhihao Yang

**Affiliations:** 1 Department of Computer Science and Engineering, Dalian University of Technology, Dalian, China

## Abstract

**Background:**

Protein complexes can be identified from the protein interaction networks derived from experimental data sets. However, these analyses are challenging because of the presence of unreliable interactions and the complex connectivity of the network. The integration of protein-protein interactions with the data from other sources can be leveraged for improving the effectiveness of protein complexes detection algorithms.

**Methods:**

We have developed novel semantic similarity method, which use Gene Ontology (GO) annotations to measure the reliability of protein-protein interactions. The protein interaction networks can be converted into a weighted graph representation by assigning the reliability values to each interaction as a weight. Following the approach of that of the previously proposed clustering algorithm IPCA which expands clusters starting from seeded vertices, we present a clustering algorithm OIIP based on the new weighted Protein-Protein interaction networks for identifying protein complexes.

**Results:**

The algorithm OIIP is applied to the protein interaction network of Sacchromyces cerevisiae and identifies many well known complexes. Experimental results show that the algorithm OIIP has higher F-measure and accuracy compared to other competing approaches.

## Background

In the post-genomic era, one of the most important issues is to systematically analyze and comprehensively understand the topology of biological networks and biochemical progress in cells. The current knowledge base of protein-protein interactions has been built from the heterogeneous data sources generated by high-throughput techniques [1-4].Protein complexes can help us to understand certain biological progress and to predict the functions of proteins. A wide range of graphtheoretic approaches have been employed for detecting protein complexes from protein interaction networks. However, they have been limited in accuracy due to the presence of unreliable interactions and the complex connectivity patterns of the networks. The experimental data sets are susceptible to false positives, i.e., some fraction of the putative interactions detected must be considered spurious because they cannot be confirmed to occur in vivo[5].

To resolve the inaccuracy resulting from false connections, other functional knowledge can be integrated into the protein interaction networks. For example, many groups [6-8] have investigated the integration of gene expression data from microarray experiments to improve protein complexes identification. However, gene expression data are also susceptible to experimental sources of bias and noise. The correlations of mRNA levels with even cognate protein expression may be modest at best. These factors limit the usefulness of microarray data for assessing the reliability of protein-protein interactions. Gene Ontology (GO) [9] is another useful data source to combine with the protein interaction networks. The GO is currently one of the most comprehensive and well-curated ontology databases in the bioinformatics community. It provides a collection of well-defined biological terms, called GO terms, spanning biological processes, molecular functions and cellular components. The GO has been used to facilitate the analysis of gene expression data [10-12].

In this work, we integrate protein-protein interactions with the information content in the GO annotation database and topology weights to enhance the modularization of interaction networks. An unweighted protein interaction network can be converted into a weighted graph representation by assigning a weight to each interaction [13]. The weight of each interaction is interpreted as its reliability, i.e., the probability of the interaction being a true positive. We propose a novel method to measure the reliability of protein-protein interactions using GO annotation data and topology weights. Following the approach of that of the previously proposed clustering algorithm IPCA[14] which expands clusters starting from seeded vertices, we present a clustering algorithm OIIP based on the new large weighted protein interaction networks.

## Methods

### Weighted network

Weights quantify the likelihood of the interaction between every pair of proteins, and they can be estimated by encoding the proteins using gene ontology (GO) consortium. “Ontology” is a specification of a conceptualization that refers to the subject of existence. GO is established by the following three criteria: (I) biological process referring to a biological objective to which the gene or gene product contributes; (II) molecular function defined as the biochemical activity of a gene product; (III) cellular component referring to the place in the cell where a gene product is active. It is very common for the same protein or proteins in the same subfamily to form protein complexes, for example, protein Ste2p and Ste3p from a complex that is among activated G protein-coupled receptors in yeast cellular mating.[15] It is also common for proteins in heterofamilies to form protein complexes if they share a conservative motif, for example, protein Ctf19, Mcm21, and Okp1 from a heterocomplex in the budding yeast kinetochore.[16] Complicated protein complexes may be formed by multiple proteins, some of which share same biological processes and some are from the same subfamily, for example, Dsl1p complex, involved in Golgi-ER retrograde transport, includes Dsl1p, Dsl3p, Q/t-SNARE proteins, and so forth.[17] Thus GO consortium is considered to be a very helpful vehicle for investigating protein-protein interactions,[18] because these three criteria reflect the attribute of gene, gene product, gene-product groups and the subcellular localization[19-21].

Semantic similarity has been used in Information Science to evaluate the similarity between two concepts in a taxonomy[22], and we applied it to protein-protein interactions to estimate the similarity between two proteins. Based on the previous method [23], we proposed our semantic similarity method. We define an annotation size of a GO term as the number of annotated proteins on the GO term. The semantic similarity between two proteins is then calculated based on the annotation size of the GO term, on which both proteins are annotated. According to the transitivity property of GO annotation, if a protein x is annotated on a GO term g_i_, it is also annotated on the GO terms on the path from g_i_ to the root GO term in the GO structure. Thus, the proportion of the annotation size of a GO term to the total number of annotated proteins can quantify the specificity of the GO term. If two proteins are annotated on a more specific GO term and have more common GO terms, then they are functionally more similar.

Suppose a protein x is annotated on m different GO terms. S_i_(x) denotes a set of annotated proteins on the GO term g_i_, whose annotation includes x, where 1≤i≤m. In the same way, suppose both x and y are annotated on n different GO terms, where n≤m. S_j_(x, y) denotes a set of annotated proteins on the GO term g_j_, whose annotation includes x and y, where 1≤j≤n. Then, the minimum size of S_i_(x), min_i_|S_i_(x)|, is less than or equal to min_j_|Sj(x, y)|.C(x,y) denotes the sets of GO terms, whose annotation includes x and y. |C(x,y)| is the number of common GO terms which x and y both have.

Suppose the size of annotation represents the number of annotated proteins on a GO term. Using the annotation size of the most specific GO term, on which two proteins x and y are annotated, we define semantic similarity S_sem_(x, y) between x and y as follows:(1)

S_max_ is the maximum size of annotation among all GO terms in a DAG structure. If two proteins x and y are annotated on a more specific GO term and more common GO terms than x and z, then x is semantically more similar to y than z.

Considering the graph topology, we also involve the topology weight. For an input graph G = (V, E), we assign the topology weight of an edge [u, v] to be the number of neighbors shared by the vertices u and v. Then we assigned the sum of S_sem_(u, v) and topology weight to the edge between u and v as a weight.

### Weighted vertex and selecting seed

We define the weight of each vertex to be the sum of the weights of its incident edges. After all vertices are assigned weights, we also sort in non-increasing order the vertices by their weights and store them in a queue Sq (vertices of the same weight are ordered in terms of their degrees). The complexity of calculating edge weights and vertex weights is O(|V||E|), and the complexity of sorting all vertices by their weights is O(|V| log |V|).

The notion that vertex weight is a good measure for selecting seeds has been adopted by DPClus [40] and MCODE[24]. Here, we also pick the highest weighted vertices as the seeds. Our procedure proceeds as follows. We pick the first vertex in the queue Sq and use it as a seed to grow a new cluster. Once the cluster is completed, all vertices in the cluster are removed from the queue Sq and we pick the first vertex remaining in the queue Sq as the seed for the next cluster. There is an important difference between this seed selection procedure and the one used in the IPCA algorithm [14]. Our procedure computes the vertex weight for each vertex based on the weighted networks; while the IPCA algorithm computes the vertex weight based on the original networks. We feel that our approach is biologically more meaningful because a complex is not only a dense structure in the original protein network but also have biological function.

### Extending cluster

We introduce a new concept to measure how strongly a vertex v is connected to a subgraph K: the interaction probability E_vk_ of a vertex v to a subgraph K, where v∉K, is defined by(2)

Where e_vk_ is the sum of the weights of edges between the vertex v and K, and w_k_ is the sum of weights of edges in K. We discuss the relationship between the parameter E_vk_ and IN_vK_ introduced in the algorithm IPCA[14]. According to [14], IN_vK_ is defined as , where m_vK_ is the number of edges between the vertex v and K, and n_K_ is the number of vertices in K. By the expressions, our parameter E_vk_ is similar to the parameter IN_vK_. While our parameter considers with the biological weights, it have more biological meaning.

A cluster K is extended by adding vertices recursively from its neighbors according to the priority. The priority of a neighbor v of K is determined by the value E_vk_. This procedure is similar to the one proposed in IPCA [14], except that we do not use IN_vk_ to judge the extending. So whether a high priority vertex v is added to the cluster is determined by the Extend-judgment test below.

Let T_in_ be a threshold ranging between 0 and 1, let d be a positive integer, and let K be a subgraph. SP is the shortest path. A vertex v∉K is added to the cluster if the following two conditions are satisfied (where K + v denotes the subgraph induced by K and v):

1. E_vk_≥T_in_; and

2. The(SP(K+v)≤d)

Only when the candidate vertex v is satisfied the conditions, can it be added to the cluster. Once the new vertex v is added to the cluster, the cluster is updated.

## Evaluation

Before we present the results of our comparative experiments, let us first introduce the various evaluation metrics that have been used to evaluate their computational methods for complex detection. We will then present the experimental results of comparing different state-of-the-art techniques using these evaluation metrics.

Overall, there are three types of evaluation metrics used to evaluate the quality of the predicted complexes and compute the overall precision of the prediction methods.

### Precision, recall and f-measure

Precision, recall and F-measure are commonly-used evaluation metrics in information retrieval and machine learning. For evaluating protein complex prediction, we need to define how well a predicted complex which consists of a set of protein members, matches an actual complex, which is another set of protein members. The neighborhood affinity score NA (p, b) between a predicted complex p =(V_p_,E_p_ ) and a real complex b =(V_b_,E_b_ ) in the benchmark complex set, as defined in equation (3) below, can be used to determine whether they match with each other. If NA (p, b) ≥ ω ,they are considered to be matching (ω is usually set as 0.20 or 0.25). Let P and B denote the sets of complexes predicted by a computational method and real ones in the benchmark, respectively. Let N_cp_ be the number of predicted complexes which match at least one real complex and N_cb_ be the number of real complexes that match at least one predicted complex. Precision and Recall are then defined as follows: [25-27](3)(4)(5)(6)

F-measure, or the harmonic mean of Precision and Recall, can then be used to evaluate the overall performance(7)

### Sensitivity, positive predictive value and accuracy

Recently, sensitivity (Sn), positive predictive value (PPV) and accuracy (Acc) have also been proposed to evaluate the accuracy of the prediction methods [28,29]. Given n benchmark complexes and m predicted complexes, let T_ij_ denote the number of proteins in common between i_th_ benchmark complex and j_th_ predicted complex. Sn and PPV are then defined as follows:(8)(9)

Here N_i_ is the number of proteins in the i_th_ benchmark complex.(10)

Generally, high Sn values indicate that the prediction has a good coverage of the proteins in the real complexes, while high PPV values indicate that the predicted complexes are likely to be true positives. As a summary metric, the accuracy of a prediction, Acc, can then be defined as the geometric average of sensitivity and positive predictive value,(11)

### P-values (functional homogeneity)

As we gained more and more biological knowledge about the proteins, we can associate a protein with (possibly multiple) functional annotations. The statistical significance of the occurrence of a protein cluster (predicted protein complex) with respect to a given functional annotation can be computed by the following hypergeometric distribution in equation (12) [30,31]:(12)

Where a predicted complex C contains k proteins in the functional group F and the whole PPI network contains |V| proteins. The functional homogeneity of a predicted complex is the smallest p-value over all the possible functional groups. A predicted complex with a low functional homogeneity indicates it is enriched by proteins from the same function group and it is thus likely to be true protein complex. By setting a common threshold which specifies the acceptable level of statistical significance, the numbers of predicted complexes with functional homogeneity under this threshold for the various methods can then be used for evaluating their respective overall performance.

## Results and discussion

The protein interaction database is downloaded from the Gavin database [31] and BioGrid (version yeast HC-BIOGRID-2.0.31). The protein-complex dataset CYC2008 [38] which we used is a comprehensive catalogue of 408 manually curated heterometic protein complexes reliably backed by small-scale experiment reported. We apply the proposed algorithm OIIP to this two databases. In the following subsections, we discuss the effect of the value T_in_ on clustering, compare the predicted clusters with the known complexes, evaluate the significance of the predicted clusters. We will also compare the algorithm OIIP to eight competing previous methods for their performance of identifying protein complexes. Since most proteins in the same complex have same or correlative function and involve in the same biological process, we employ biological annotation information, including Go cellular component annotation [39], GO Molecular Function annotation [39] and GO Biological process annotation [39] to assess the predicted protein-complexes.

### The effect of T_in_ on clustering

**Figure 1:**
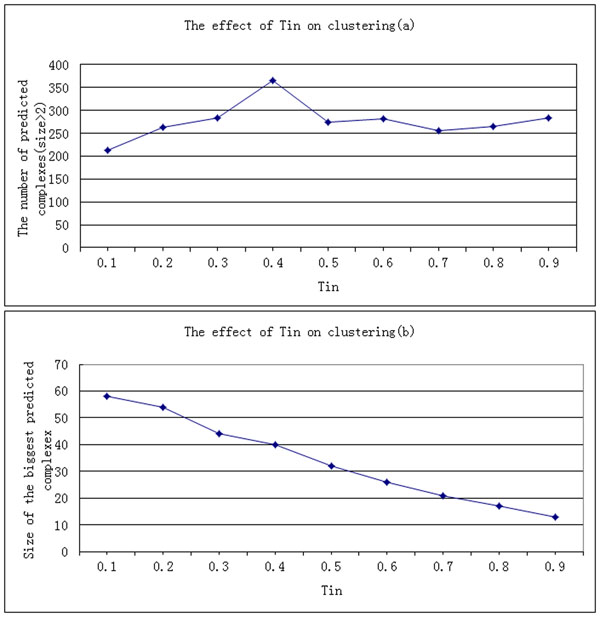
**The effect of T_in_ on clustering.** The effect on the predicted clusters with different T_in_ is given in this Figure 1. Figure 1(a) shows that the total number of the predicted clusters is increasing as T_in_ increases. However, there is an abrupt decrease at T_in_ = 0.5. Figure 1(b) shows that the size of the biggest cluster is decreasing as T_in_ increases. With the increasing of T_in_, the probability of neighbors added to the cluster is decreasing. Thus, the size of the predicted clusters is also decreasing.

To understand how the value of T_in_ influences the outcome of the clustering, we generate 9 sets of clusters by using T_in_ = 0.1, 0.2,..., 0.9 from the Gavin PPI dataset. The effect on the predicted clusters with different T_in_ is given in Figure 1.

Figure 1(a) shows that the total number of the predicted clusters is increasing as T_in_ increases. However, there is a abrupt decrease at T_in_ = 0.5. This is probably caused by the Hub structures in the protein interaction network. When T_in_ = 0.5, these Hub structures are decomposed into complexes that consist of only 2 proteins. Figure 1(b) shows that the size of the biggest cluster is decreasing as T_in_ increases. With the increasing of T_in_, the probability of neighbors added to the cluster is decreasing. Thus, the size of the predicted clusters is also decreasing.

As shown in Table 1, the Precision of the algorithm OIIP is about 0.6 when T_in_=0.5, which implies that the clusters generated by OIIP are reliable. The F-measure is about 0.45 when T_in_=0.4, which represents overall performance of an algorithm. The Accuracy takes into account of both the sensitivity and PPV, and is determined by the larger one. In this experiment, the Accuracy is mostly influenced by the PPV. The PPV of the clusters generated by OIIP increases with the increasing of T_in_. Especially, an obvious increase appears when T_in_ ≥ 0.5.

**Table 1:** The Precision, Recall, F-measure, sensitivity, PPV and Accuracy of the predicted complexes by OIIP using different parameters

Parameter	Precision	Recall	F-measure	sensitivity	PPV	Accuracy
T_in_=0.1	0.4819	0.335784	0.395787	0.525	0.465604	0.494491
T_in_=0.2	0.498113	0.352941	0.413146	0.511979	0.48535	0.498487
T_in_=0.3	0.482112	0.365196	0.415588	0.501042	0.516012	0.508472
T_in_=0.4	0.559816	0.384804	0.456097	0.484896	0.560703	0.521424
T_in_=0.5	0.606195	0.335784	0.432177	0.420312	0.57216	0.490394
T_in_=0.6	0.582645	0.340686	0.429963	0.409375	0.621008	0.504207
T_in_=0.7	0.485768	0.343137	0.402181	0.386458	0.638547	0.496761
T_in_=0.8	0.458477	0.338235	0.389283	0.360417	0.653266	0.48523
T_in_=0.9	0.466227	0.345588	0.396944	0.340625	0.663043	0.475236

### Comparison of OIIP and other methods

**Figure 2:**
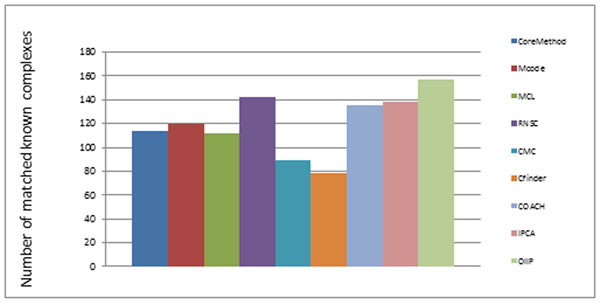
**Comparison of the predicted clusters with the known complexes on Gavin PPI dataset.** Figure 2 shows the numbers of known complexes (size>2) matched to the clusters generated by OIIP and by other eight previous known methods: CoreMethod[15], Mcode[9], MCL[13], RNSC[12], CMC[14], CFinder[11], COACH[10], IPCA[5].

Since there have been protein complexes that were experimentally determined, a good protein complexes detecting algorithm should identify these known complexes as many as possible. Figure 2 shows the numbers of known complexes (size>2) matched to the clusters generated by OIIP and by other eight previous known methods: CoreMethod[37], Mcode[24], MCL[34], RNSC[33], CMC[36], CFinder[32], COACH[35], IPCA[14]. Our method predicted more complexes than others on Gavin PPI dataset. As show in Table 2 OIIP also get the highest F-measure (T_in_=04). Although Mcode has the highest precision, its recall is very low. The fact is that the predicted protein complexes are fewer than our method. Some predicted clusters which are not matched with criterion complexes are possible actual complexes which are undiscovered. So it is necessary to predict more clusters with high F-measure value.

**Table 2:** Performance comparison of Identify protein complexes methods on Gavin dataset

Method	Precision	Recall	F-measure	Accuracy	P-Value		
	
					GO_Function	GO_Process	GO_Component
CoreMethod	0.526596	0.279412	0.365101	0.491702	0.579787	0.430851	0.478723
Mcode	0.733333	0.294118	0.419847	0.469031	0.511111	0.392593	0.474074
MCL	0.540373	0.27451	0.364071	0.509139	0.503106	0.391304	0.459627
RNSC	0.400651	0.348039	0.372497	0.489759	0.332248	0.247557	0.309446
CMC	0.608	0.218	0.3211	0.474	0.692	0.55	0.633
CFinder	0.663	0.191	0.297	0.419	0.602	0.439	0.551
COACH	0.524	0.331	0.406	0.49	0.656	0.525	0.61
IPCA	0.526032	0.338235	0.411731	0.48789	0.719928	0.587074	0.666068
OIIP T=0.4	0.559816	0.384804	0.456097	0.521424	0.697853	0.553681	0.636503
OIIP T=0.5	0.606195	0.335784	0.432177	0.490394	0.769912	0.639381	0.710177

And we count the number of clusters with p-value less than 0.01, a threshold which represents significant biological sense and compute the proportion of clusters which achieve low p-value. The proportion of clusters from various methods with low p-value is shown in Table 2. Table 2 also shows that the clusters predicted by our method have achieved highest biological significance than predicted clusters from others on all the three biological annotation datasets when T is set to 0.5. Compare to the IPCA, we have better performance in all evaluation measurements. So the ontology interaction to the PPI network is valuable to predict protein complexes.

**Figure 3:**
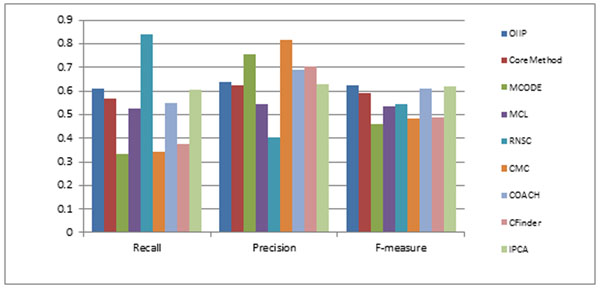
**The Precision, Recall and F-value of results predicted from various methods on BioGrid PPI dataset. **As show in Figure 3, RNSC has the highest recall and CMC has the highest precision, while our method OIIP also gets the highest F-measure on BioGrid PPI dataset.

As show in Figure 3, RNSC has the highest recall and CMC has the highest precision, while our method OIIP also gets the highest F-measure on BioGrid PPI dataset.

### Examples of predicted complexes

From the results of the experiment above we know that most of our predicted clusters have highly biological meanings. We give some examples of predicted clusters with detailed information which are matched with the benchmark complexes in Table 3. We also list the best matched benchmark complexes, NA and the p-value of three protein annotation.

**Table 3:** Some predicted clusters which matched with benchmark complexes

ID	Predicted clusters	Benchmark complexes ID in CYC2008	NA	p-value		
	
				GO_Component	GO_Function	GO_Process
1	YBR123C YOR110W YPL007C YDR362C YAL001C YGR047C	402	1	0	3.16E-06	2.59E-05

2	YLR208W YGL092W YDL116W YJR042W YKL057C YGL100W	181	1	1.10E-05	2.50E-06	3.81E-06

3	YLR166C YBR102C YPR055W YIL068C YER008C YDR166C YGL233W YJL085W	90	1	2.68E-06	2.06E-04	4.37E-03

4	YBR234C YLR370C YJR065C YDL029W YIL062C YNR035C YKL013C	12	1	1.19E-03	4.95E-06	9.54E-07

5	YER157W YGR120C YPR105C YNL051W YML071C YGL223C YNL041C YGL005C	112	1	1.05E-05	6.91E-02	2.50E-06

6	YHR081W YHR069C YOL021C YGR095C YGR195W YDR280W YGR158C YCR035C YDL111C YNL232W YOR001W YOL142W YOR076C	178	0.923077	3.00E-03	0	0

7	YGL048C YKL145W YHR027C YHL030W YLR421C YHR200W YDR427W YDL147W YFR052W YDL097C YPR108W YIL075C YFR004W	2	0.911157	1.09E-05	2.68E-06	4.17E-06

8	YPL210C YDL092W YML105C YPR088C YPL243W YDL051W YKL122C	248	0.857143	3.46E-06	1.19E-07	1.19E-07

9	YOR179C YDR195W YGR156W YER133W YAL043C YKL059C YPR107C YLR115W YDR301W YNL317W YKR002W YKL018W YLR277C	341	0.816667	9.43E-04	0	2.32E-06

10	YMR223W YGL066W YBR081C YGR252W YDR448W YGL112C YDR145W YMR236W YDR167W YBR198C YOL148C YLR055C YPL254W YDR392W YCL010C YDR176W YHR099W YML007W	227	0.802778	1.39E-04	0	0

**Figure 4:**
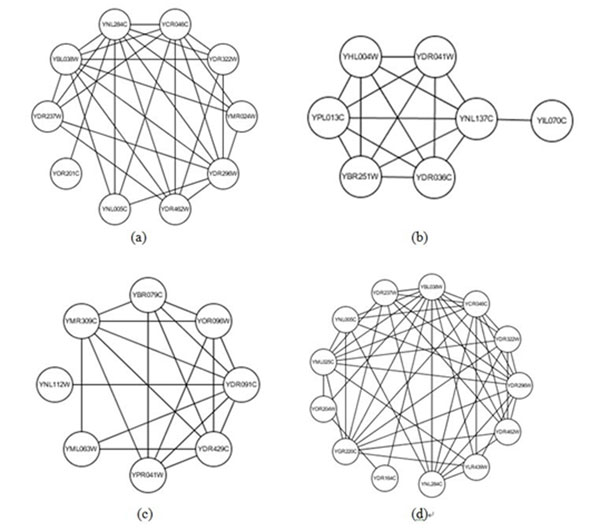
**Some predicted complexes which don’t match with benchmark complexes.** Some of predicted clusters from our method are not matched with complex from criterion dataset. But we find that they have highly biologically significant and have high local density, so some of them may be real complexes which are still undiscovered. We also give some examples in Figure 4. Their p-value of biological annotation shows that some of them may be the candidate protein complexes. The results are useful for biologists to find the new protein complexes.

Some of predicted clusters from our method are not matched with complex from criterion dataset. But we find that they have highly biologically significant and have high local density, so some of them may be real complexes which are still undiscovered. We also give some examples in Table 4 and Figure 4. Their p-value of biological annotation shows that some of them may be the candidate protein complexes. The results are useful for biologists to find the new protein complexes.

**Table 4:** Some predicted complexes which don’t match with benchmark complexes

ID	Main proteins	P-value		
	
		GO_Component	GO_Function	GO_Process
Complex a	YOR201C YNL284C YCR046C YBL038W YDR237W YDR462W YDR296W YDR322W YNL005C YMR024W	9.89E-06	2.32E-06	8.94E-07

Complex b	YIL070C YNL137C YHL004W YPL013C YBR251W YDR041W YDR036C	5.39E-05	8.58E-06	4.95E-06

Complex c	YNL112W YDR091C YMR309C YPR041W YBR079C YDR429C YOR096W YML063W	0	2.03E-06	3.04E-04

Complex d	YDR164C YOR204W YBL038W YML025C YGR220C YCR046C YNL005C YDR296W YDR237W YNL284C YDR322W YDR462W YLR439W	9.36E-06	9.18E-06	7.45E-06

## Conclusions

It is believed that identification of protein complexes is useful to explain certain biological progress and to predict functions of proteins. In this paper, we developed an algorithm OIIP to identify protein complexes based on the new large weighted protein interaction networks. Experimentally generated protein-protein interaction data includes an enormous amount of false positives. So we introduced a semantic similarity method to measure the reliability of interactions. For this measurement, we use the annotations in Gene Ontology (GO), which provides the comprehensive functional information. When we implemented the OIIP algorithm with weighted networks, the overall F-measure and accuracy of complexes is substantially improved. This result strongly appeals the necessity of integrating of functional information for the analysis of protein-protein interaction data.

The fact that biological properties are poor at the identification reveals that the higher-level structures (e.g., secondary and tertiary structure) of proteins cannot be accurately represented by the primary structure under the current coding techniques. The experimentally determined protein interaction network has not been used in the research, and a possible future research could combine the experimentally determined protein interactions with the GO estimated interactions to further improve the identification.

## Competing interests

The authors declare that they have no competing interests.

## Authors' contributions

Please see sample text in BX identified all protein complexes and generated this manuscript; ZHY provided data information and edited this manuscript; HFL supervised all aspects of this manuscript. All authors read and approved the final manuscript.

## References

[B1] Uetz P A comprehensive analysis of protein-protein interactions in Saccharomyces cerevisiae Nature 2000 403 1 623 627 10688190 10.1038/35001009

[B2] Ito T A comprehensive two-hybrid analysis to explore the yeast protein interactome PNAS 2001 98 8 4569 4574 10.1073/pnas.061034498 11283351 PMC31875

[B3] Gavin AC Functional organization of the yeast proteome by systematic analysis of protein complexes Nature 2002 415 1 141 147 11805826 10.1038/415141a

[B4] Ho Y Systematic identification of protein complexes in Saccharomyces cerevisiae by mass spectrometry Nature 2002 415 1 180 183 11805837 10.1038/415180a

[B5] von Mering C Comparative assessment of large-scale data sets of protein-protein interactions Nature 2002 417 1 399 403 12000970 10.1038/nature750

[B6] Cho YR Hwang W Zhang A Identification of overlapping functional modules in protein interaction networks: information flow-based approach Proceedings of 6th IEEE International Conference on Data Mining Workshops 2006 147 152

[B7] Ideker T Ozier O Schwikowski B Siegel AF Discovering regulatory and signalling circuits in molecular interaction networks Bioinformatics 2002 18 1 S233 S240 10.1093/bioinformatics/18.suppl_1.S233 12169552

[B8] Tornow S Mewes HW Functional modules by relating protein interaction networks and gene expression Nucleic Acids Res 2003 31 21 6283 6289 10.1093/nar/gkg838 14576317 PMC275479

[B9] The Gene Ontology Consortium The Gene Ontology (GO) project in 2006 Nucleic Acids Research 2006 34 1 322 326 10.1093/nar/gkj439 16381878 PMC1347384

[B10] Hvidsten TR Lagreid A Komorowski J Learning rule based models of biological process from gene expression time profiles using Gene Ontology Bioinformatics 2003 19 9 1116 1123 10.1093/bioinformatics/btg047 12801872

[B11] Fang Z Yang J Li Y Luo Q Liu L Knowledge guided analysis of microarray data Journal of Biomedical Informatics 2006 39 1 401 411 16214421 10.1016/j.jbi.2005.08.004

[B12] Doherty JM Carmichael LK Mills JC GOurmet: A tool for quantitative comparison and visualization of gene expression profiles based on gene ontology (GO) distributions BMC Bioinformatics 2006 7 151 332 365 16545118 10.1186/1471-2105-7-151 PMC1459206

[B13] Barrat A Barthelemy M Pastor-Satorras R Vespignani A The architecture of complex weighted networks PNAS 2004 101 11 3747 3752 10.1073/pnas.0400087101 15007165 PMC374315

[B14] Li Min Chen Jianer Wang Jianxin Modifying the DPClus algorithm for identifying protein complexes based on new topological structures BMC Bioinformatics 2008 9 398 345 352 18816408 10.1186/1471-2105-9-398 PMC2570695

[B15] Shi C Kaminskyj S Caldwell S Loewen MC A role for a complex between activated G protein-coupled receptors in yeast cellular mating Proc. Natl. Acad. Sci. U.S.A 2007 104 13 5395 5400 10.1073/pnas.0608219104 17369365 PMC1838501

[B16] Ortiz J Stemmann O Rank J Lechner: A putative protein complex consisting of Ctf19, Mcm21, and Okp1 represents a missing link in the budding yeast kinetochore Genes Dev 1999 13 9 1140 1155 10.1101/gad.13.9.1140 10323865 PMC316948

[B17] Kraynack BA Chan A Rosenthal E Essid M Umansky B Waters MG Schmitt HD Dsl1p, Tip20p, and the novel Dsl3(Sec39) protein are required for the stability of the Q/t-SNARE complex at the endoplasmic reticulum in yeast Mol. Biol. Cell 2005 16 9 3963 3977 10.1091/mbc.E05-01-0056 15958492 PMC1196311

[B18] Chou KC Cai YD Predicting Protein-Protein Interactions from Sequences in a Hybridization Space Proteome Res 2006 5 1 316 322 10.1021/pr050331g 16457597

[B19] Ashburner M Gene Ontology: tool for the unification of biology Nat. Genet 2000 25 1 25 29 10.1038/75556 10802651 PMC3037419

[B20] Chou JJ Li H Salvessen GS Yuan J Wagner G Solution structure of BID, an intracellular amplifier of apoptotic signalling Cell 1999 96 1 615 624 10089877 10.1016/s0092-8674(00)80572-3

[B21] Oxenoid K Chou JJ The structure of phospholamban pentamer reveals a channel-like architecture in membranes Proc. Natl. Acad. Sci., U.S.A 2005 102 1 10870 10875 10.1073/pnas.0504920102 16043693 PMC1182456

[B22] Resnik P Using information content to evaluate semantic similarity in a taxonomy The Proc. of 14th International Joint Conference on Artificial Intelligence 1995 448 453

[B23] Cho Y-R Hwang W Ranmanathan M Zhang A Semantic integration to identify overlapping functional modules in protein interaction networks BMC Bioinformatics 2007 265 8 147 160 10.1186/1471-2105-8-265 PMC1971074 17650343

[B24] Bader G Hogue C An Automated Method for Finding Molecular Complexes in Large Protein Interaction Networks BMC Bioinformatics 2003 4 2 455 470 10.1186/1471-2105-4-2 PMC149346 12525261

[B25] Chua HN Ning K Sung WK Leong HW Wong L Using indirect protein-protein interactions for protein complex prediction CSB 2007 2 1 97 109 17951816

[B26] Geva G Sharan R Identification of Protein Complexes from Co-immunoprecipitation Data Bioinformatics 2009 74 3 1223 1146 10.1093/bioinformatics/btq652 PMC3008648 21115439

[B27] Wu M Li XL Kwoh CK Ng SK A Core-Attachment based Method to Detect Protein Complexes in PPI Networks BMC Bioinformatics 2009 10 169 122 142 19486541 10.1186/1471-2105-10-169 PMC2701950

[B28] Brohee S van Helden J Evaluation of clustering algorithms for protein-protein interaction networks BMC Bioinformatics 2006 7 1 488 502 10.1186/1471-2105-7-488 17087821 PMC1637120

[B29] Friedel CC Krumsiek J Zimmer R Boostrapping the Interactome: Unsupervised Identification of Protein Complexes in Yeast RECOMB 2008 1 1 3 16 10.1089/cmb.2009.0023 19630542

[B30] Bu D Zhao Y Cai L Xue H Zhu X Lu H Zhang J Sun S Ling L Zhang N Li G Chen R Topological structure analysis of the protein-protein interaction network in budding yeast Nucleic Acids Res 2003 31 9 2443 2450 10.1093/nar/gkg340 12711690 PMC154226

[B31] Gavin AC Aloy P Grandi P Proteome survey reveals modularity of the yeast cell machinery Nature 2006 440 1 631 636 16429126 10.1038/nature04532

[B32] Adamcsek B Palla G Farkas I Derenyi I Vicsek T CFinder: locating cliques and overlapping modules in biological networks Bioinformatics 2006 22 8 1021 1043 10.1093/bioinformatics/btl039 16473872

[B33] King A Przulj N Jurisica I Protein Complex Prediction via Cost-based Clustering Bioinformatics 2004 20 1 3013 3020 10.1093/bioinformatics/bth351 15180928

[B34] Pereira-Leal JB Enright AJ Ouzounis CA Detection of functional modules from protein interaction networks Protein Structure Function, and Bioinformatics 2004 54 1 49 57 10.1002/prot.10505 14705023

[B35] Wu M Li XL Kwoh CK Ng SK A Core-Attachment based Method to Detect Protein Complexes in PPI Networks BMC Bioinformatics 2009 10 1 169 181 10.1186/1471-2105-10-169 19486541 PMC2701950

[B36] Liu GM Chua HN Wong L Complex discovery from weighted PPI networks Bioinformatics 2009 25 15 1891 1897 10.1093/bioinformatics/btp311 19435747

[B37] Leung HC Yiu SM Xiang Q Chin FY Predicting Protein Complexes from PPI Data: A Core-Attachment Approach Journal of Computational Biology 2009 16 2 133 144 10.1089/cmb.2008.01TT 19193141

[B38] Shuye Pu Wong Jessica Turner Brian Up-to-data catalogues of yeast protein complexes Nucleic Acids Research 2009 37 1 825 831 19095691 10.1093/nar/gkn1005 PMC2647312

[B39] TG Consortium The Gene Ontology Consortium: Creating the gene ontology resource: design and implementation Genome Res 2001 11 1 1425 1433 11483584 10.1101/gr.180801 PMC311077

[B40] Altaf-Ul-Amin M Shinbo Y Mihara K Kurokawa K Kanaya S Development and implementation of an algorithm for detection of protein complexes in large interaction networks BMC Bioinformatics 2006 207 3 134 163 10.1186/1471-2105-7-207 PMC1473204 16613608

